# A Deep Learning Model for Predictive Maintenance in Cyber-Physical Production Systems Using LSTM Autoencoders

**DOI:** 10.3390/s21030972

**Published:** 2021-02-01

**Authors:** Xanthi Bampoula, Georgios Siaterlis, Nikolaos Nikolakis, Kosmas Alexopoulos

**Affiliations:** Laboratory for Manufacturing Systems and Automation, Department of Mechanical Engineering and Aeronautics, University of Patras, 26504 Patras, Greece; baboula@lms.mech.upatras.gr (X.B.); siaterlis@lms.mech.upatras.gr (G.S.); nikolakis@lms.mech.upatras.gr (N.N.)

**Keywords:** cyber-physical production systems, deep learning, artificial intelligence, Long Short-Term Memory (LSTM), predictive maintenance, remaining useful life

## Abstract

Condition monitoring of industrial equipment, combined with machine learning algorithms, may significantly improve maintenance activities on modern cyber-physical production systems. However, data of proper quality and of adequate quantity, modeling both good operational conditions as well as abnormal situations throughout the operational lifecycle, are required. Nevertheless, this is difficult to acquire in a non-destructive approach. In this context, this study investigates an approach to enable a transition from preventive maintenance activities, that are scheduled at predetermined time intervals, into predictive ones. In order to enable such approaches in a cyber-physical production system, a deep learning algorithm is used, allowing for maintenance activities to be planned according to the actual operational status of the machine and not in advance. An autoencoder-based methodology is employed for classifying real-world machine and sensor data, into a set of condition-related labels. Real-world data collected from manufacturing operations are used for training and testing a prototype implementation of Long Short-Term Memory autoencoders for estimating the remaining useful life of the monitored equipment. Finally, the proposed approach is evaluated in a use case related to a steel industry production process.

## 1. Introduction

Digitization in production systems mandates the use of new Information and Communication Technology (ICT) systems in order to improve performance in manufacturing. However, the enormous amount of information generated and gathered by manufacturing ICT systems as well as Internet of Things (IoT) devices installed on the factory floor usually remains underutilized. New methods and models are needed that can truly benefit the ICT landscape and improve production processes [1,2]. Considering that a cyber-physical system (CPS) can be defined as a closed-loop control system, coupling physical assets to software modules [3], the application of its principles to a production system denote a cyber-physical production system, or CPPS [4,5]. In contrast to the traditional automation pyramid [6], a CPPS consists of many distributed and interconnected systems managing or controlling different aspects of manufacturing processes. This may vary from simple monitoring to planning, control, and online reconfiguration of a production system. The use of containerization technologies towards holistic orchestration and control of a CPPS is discussed in Nikolakis et al. [7].

Furthermore, the evolution of embedded systems and sensors in conjunction with the ever-increasing digitization of modern shop-floors has enabled the generation of an enormous volume of digital information. The analysis of those data may reveal underlying patterns not visible to the human operator and may support proactive decision making [8]. Hence, insight can be created on the actual condition of production equipment, through the adoption of data-driven techniques for condition monitoring and assessment of its operational condition, as discussed in Entezami et al. [9] and Chuang et al. [10]. In turn, this can enable a transition from time-based preventive maintenance to predictive maintenance (PdM) or a combination of them. This can reduce maintenance, and thus production costs, by assessing the current condition of the equipment and estimating its remaining useful life (RUL). Towards that end, the employment of artificial intelligence (AI) techniques, and in particular machine learning (ML) approaches, capable of analyzing large-scale data sets and detecting underlying patterns, can enable proactive decision-making, such as in the context of predictive maintenance [11].

A novel approach for prediction and fault detection is proposed in this work. It relies on autoencoders with Long Short-Term Memory (LSTM) networks to assess the operational condition of production equipment, using a deep learning method for anomaly detection that is then mapped to different RUL values. A combination of more than one network is proposed for classifying the current machine’s health condition to one or more corresponding labels. The main novelty of this approach is that a separate neural network is trained for each label leading to better results for each case. Consequently, this method can be adjusted to several types of machines and labels. The proposed approach relies on the reconstruction error that the LSTM-autoencoders commit trying to reconstruct data values the network has never seen in the training phase, and it has also been evaluated in an industrial case based on historical maintenance record datasets. Finally, the development of a prototype method and the implementation of a software prototype have shown that the proposed method can provide information regarding the machine’s health without requiring any specialization and additional skills from the industry operators.

## 2. Literature Review

Condition monitoring of industrial equipment has attracted heightened interest in the last decade [12,13,14,15]. As a consequence and empowered by the progress in AI, predictive analytics emerged for identifying abnormalities in large-scale datasets and, among others, determining imminent maintenance needs [16]. The adoption of AI, and in particular ML, methods for PdM purposes has the potential to prevent equipment failures, without requiring a clear understanding of the production process itself or the collected data [17,18]. The reason is that data-driven approaches can be used to train ML models with run-to-failure data without requiring knowledge of the underlying process [19,20].

Several ML techniques have been investigated throughout the years. An overview of ML approaches for predictive maintenance is presented in Carvalho et al. [21] and Zonta et al. [22]. Artificial neural network combined with data mining tools [23] and Bayesian networks [24] was used for large manufacturing datasets to diagnose and predict faults, nonetheless presenting issues associated with process time and the computational learning aspects, respectively, due to the large amount of data. In addition, for sequence-to-sequence learning, transformer models have recently received increased attention as discussed in Wu et al. [25] and Vaswani et al. [26].

In particular, Convolutional Neural Networks (CNNs) are suggested for fault diagnosis over multi-channel data from sensors with excellent performance and lower computational cost, requiring homogeneity of the multi-channel data [27]. In order to overcome this problem, a Double Deep Autoencoder structure is proposed in Chen and Huang [28] for clustering distributed and heterogeneous datasets. Autoencoders consist of exactly one input and output layer and one or more hidden layers. The input values are compressed at the encoding stage, and then the same values are reconstructed at the decoding stage [29].

Concurrently, for sequential data such as time-series data, Recurrent Neural Networks (RNNs) are considered more suitable than CNNs [30,31]. RNNs contain feedback loops and have the ability to remember the information of former units. Although they are capable of capturing long-term temporal dependencies from the data, they have restrictions on long-term RUL predictions [32]. Small gradients tend to slowly shrink and eventually disappear during propagation across multiple unfoldings of network layers [33]. Popular variants of RNNs that avoid these limitations are Long Short-Term Memory (LSTM) networks and Gated Recurrent Unit (GRU) networks [31,34,35,36]. LSTM hidden structure includes a new unit, a memory cell, capable of representing the long-term dependencies in sequential time-series data contrary to GRU networks [37,38,39,40]. Nevertheless, because of sensitivity to dataset changes or program tuning parameters, internal layers and cells in LSTM networks seem to lack efficiency [41].

LSTM memory cells consist of different neural networks, called gates. Gates control the interactions between the memory units and decide what data are relevant to keep or forget during training. The input gate is responsible to decide if the state of the memory cell can be modified by the input signal, while the output gate determines if the state of other memory cells can be modified by the input signal. Finally, the forget gate is in charge of deciding whether to forget or remember the previous status of the signal [42,43].

LSTM-autoencoders are capable of dealing with data sequences as input in contrast to regular autoencoders. Many studies have presented LSTM-autoencoders as a promising tool for time series predictions. LSTM-autoencoder was used for traffic flow prediction and had an outstanding performance not only mining deeply big data considering the temporal characteristics but also capturing the spatial characteristics of traffic flow in comparison with CNN and SVM models [44]. Stacked autoencoders with LSTM were used to predict the one-step-ahead closing price of six popular stock indices traded in different financial markets, and they outperformed WLSTM (i.e., a combination of WT and LSTM), LSTM, and the conventional RNN in both predictive accuracy and profitability [45]. Moreover, autoencoders combined with LSTM presented as the best-performing model in terms of RMSE values for different training and test data sets. This shows the better capability of feature extraction of these models enabling better forecast than multilayer perceptron, deep belief network, and single LSTM [46]. Consequently, the combination of autoencoders and LSTM has shown high potential in time series prediction.

The purpose of a deep learning LSTM-autoencoder network is to gather and extract composite information from large time-series datasets using many hidden layers [47]. However, choosing suitable hyperparameters is a complex task and significantly affects the model’s performance [48]. For example, more hidden layers or more neurons in a network does not necessarily increase the performance of the network. This hyperparameter selection depends on different factors such as the amount of data or the generating process [49].

Consequently, this paper proposes an LSTM-based autoencoder model for estimating the health state of an industrial machine. Autoencoder neural networks are preferred in order to learn a compressed representation of input data and change the input data dimension, while the LSTM network is considered suitable for processing time-series data and identifying their temporal patterns. The proposed LSTM-autoencoder consists of an autoencoder for sequential data combined with an LSTM network [50]. The LSTM encoder is trained on historical time-series data and records from previous maintenance activities to produce a fixed-length vector that is imported to the decoder, which in turn classifies it in one of the predetermined labels. After the classification step, the outcome is associated to a RUL value enabling predictive maintenance actions to take place.

## 3. Approach

**Figure 1:**
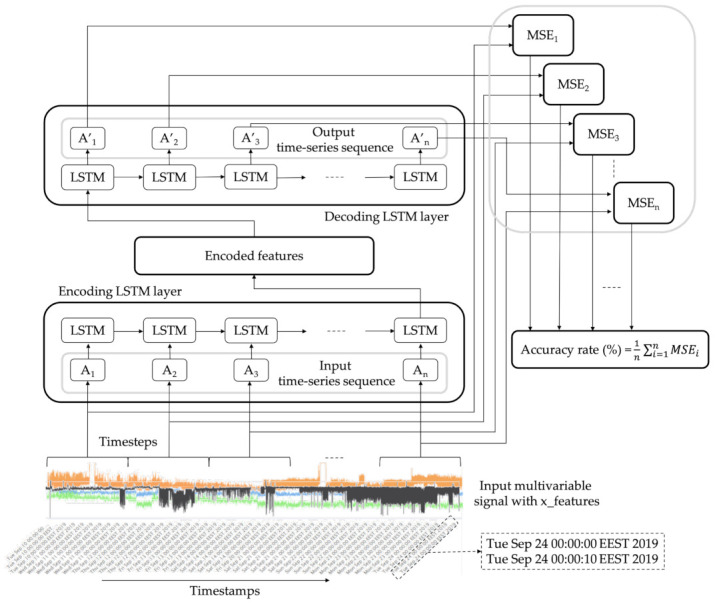
High-level Long Short-Term Memory (LSTM)-autoencoder architecture.

This study investigates a supervised deep learning approach to estimate the health status of a machine. Sensor data, monitoring different parameters of a production machine, are acquired and used to train a set of LSTM-autoencoders. At the next step, the trained LSTM-autoencoders can classify new streams of incoming data to different operational status. A high-level architecture illustrating the proposed concept is provided in Figure 1.

After the classification, an estimation of the RUL value can be determined based on the input dataset’s performance. This, depends among others, on the time that the machine required for issuing a replacement order in the past that presented similar values to the input.

In particular, real-world data are acquired via sensors that are placed on the machine, measuring key process-related features from the equipment and its environment, the processing of which will generate the required features for enabling the proposed learning procedure. Nevertheless, it is challenging to identify the correct set of features that may be associated to potential failures. Critical values should be determined and modeled considering the degradation process of the machine. Hence, the key features used for the analysis are selected using knowledge stemming from the machine’s related studies and process experts that were acquired in the context of this work.

LSTM-autoencoders are used for classifying the current machine’s health condition to one or more corresponding labels. The architecture of each LSTM-autoencoder depends on the nature of the problem [51,52]. At a minimum, two labels are required, one determining the “good” condition of the equipment, usually referring at (1) a time right after a maintenance activity or a part replacement has occurred, and (2) a label characterizing a failure, an alarm or a low health-level, from an operational perspective, mandating for maintenance to be carried out. Apart from the two aforementioned categories, more could be added depending on a case-by-case analysis and requirements. In this study, three labels have been used to determine a high, medium, and low level of equipment health status. The high and low levels, named as “good” and “bad” labels in the present study, correspond to an observation window right before and after the emergence of restoration activity, bringing back the machine to its normal operational condition. This may or may not include a breakdown of the machine.

For each label, a single LSTM-autoencoder is trained, so as the training dataset includes only the temporal data related with the corresponding machine’s status. The input of each LSTM-autoencoder is a time-series sequence, denoted in this work as *A*_*i*_ with $\alpha_{i_{j}}$ being the values of the sensors denoting one of the variables measured at a specific time, with n being the number of features, as presented in Equation (1).

(1) $$A_{i}=\left[\alpha_{i_{1}},\alpha_{i_{2}},\alpha_{i_{3}},...,\alpha_{i_{j}}\right],\;\mathrm{where}\;\alpha_{i_{j}}\in R,\;\mathrm{with}\;i,\;j\in Z\;\mathrm{and}\;i\;\leq \;n$$

Each time-series sequence is imported into a new encoder LSTM cell together with the hidden output from the previous LSTM cell. The hidden output from the last LSTM cell of the encoder is encoded finally into a learned representation vector. This vector may be an entire sequence of hidden states from all the previous encoder LSTM cells.

Then, the decoder takes the encoded features as input in order to be processed through the various LSTM decoder cells and finally produce the output. The output of the decoder layer is a reconstruction of the initial input time-series sequence, represented as $A^{\prime }$_*i*_, with the reconstructed values of the sensors, $\alpha_{i_{j}}^{\prime }$, presented in Equation (2).

(2) $$A_{i}^{\prime }=\left[\alpha_{i_{1}}^{\prime },\alpha_{i_{2}}^{\prime },\alpha_{i_{3}}^{\prime },...,\alpha_{i_{j}}^{\prime }\right],\;\mathrm{where}\;\alpha_{i_{j}}^{\prime }\in R,\;\mathrm{with}\;i,\;j\in Z\;\mathrm{and}\;i\;\leq \;n$$

This requires out of the entire set of sensor data to separate them in order to properly train each autoencoder. This is possible by using the actual maintenance records and expert knowledge. In greater detail, past maintenance records contain the dates and causes requiring maintenance. This could also be provided by the preventive maintenance plans set in place by the equipment owner. This information, in combination with historical sensor data values from these specific dates, make it possible to label the data values and distinguish the datasets for network training and testing. Data values are categorized and separated according to the number and kind of statuses chosen, based on their timestamp. Then, in order to define, train, and test data for each LSTM-autoencoder, a simple split is performed in each dataset; 90% of the first part of the dataset is the train data, and the remaining 10% is the test data.

The accuracy of a model is usually determined after the model training. Test samples are fed as input to the model, and the network compares the initial input values with the reconstructed ones. The mean squared error (MSE) of the difference between the reconstructed time-series sequence, $A^{\prime }$_*i*_, and the initial input, *A*_*i*_, is the cost function of the LSTM-autoencoder, as presented in Equation (3).

(3) $$MSE_{i}=\frac{1}{n}\sum_{i=1}^{n}{\left(A_{i}^{\prime }-A_{i}\right)}^{2}$$

For each time-sequence, a mean squared error is calculated. Then, the accuracy rate (%) of the LSTM-autoencoder is obtained from the average calculation of these values.

After the training of the set of LSTM-autoencoders, the same dataset becomes the input to each of these trained networks. Then, according to the accuracy rate that appears with this input, classification of the input dataset is possible.

As mentioned before, in this study three labels have been used to determine a high, medium, and low level of equipment health status. As a consequence, three LSTM-autoencoders should be trained, and each of them with a dataset includes only the temporal data related with the corresponding machine’s status. So, for example, if the accuracy rate is bigger at the LSTM autoencoder trained with the data from the healthy status of the machine, then the input unknown dataset also contains data values that correspond to a healthy machine status.

## 4. Implementation

In order to test and validate the potential contribution of the proposed approach for future real-world applications, the aforementioned method has been implemented into a prototype software system using Python 3.7 [53].The resources used in order to integrate the aforementioned system were a computer with an Intel i7 processor (Intel(R) Core(TM) i7-3770 CPU @3.40 GHz 3.80 Ghz), (Intel, Santa Clara, CA, USA), regarding the processing power, and an eight (8) gigabyte RAM memory (Samsung, Seoul, Korea). The operating system that the proposed system was hosted and tested on was Microsoft Windows 10.

**Figure 2:**
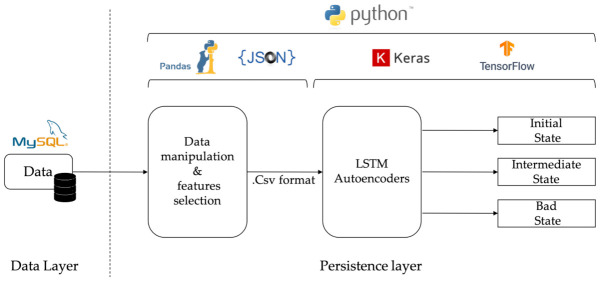
LSTM-autoencoder implementation.

The time required for the previous set up to perform the proposed approach was measured with the time() function in Python. It took one hour (1 h) for the training parameters of each LSTM-autoencoder during the training process with about 180,000 data values. Considering that, in a real-world production, RUL prediction will take place twice a day. Assuming that in a period of one (1) day there will be about 40,000 data values from the machine, the RUL prediction for a dataset of 20,000 measured 0.3 s for each LSTM-autoencoder. A high-level representation of the system’s architecture is illustrated in Figure 2.

Machine or sensor data are imported to the implemented system as JSON files via the Python data analysis library. Afterwards, imported data are processed to remove zero values and converted to a dataframe format, using the Pandas library. Each column of this dataframe includes the values of a single sensor sorted according to their timestamp. The feature selection for determining the degradation level is mostly based upon human knowledge of the equipment and process. This consists of the first set of parameters that are used for the analysis. At a second level, this set is experimentally enhanced with additional parameters to increase its performance in terms of classification accuracy to one or more labels. In this work, three labels are used identifying the good, bad, and intermediate operating condition of the monitored equipment.

The LSTM-autoencoders are implemented using Keras, a Python library for developing and evaluating deep learning models. In particular, three individual LSTM-autoencoders are trained with data corresponding to each state: initial, intermediate, and bad. The segmentation of the training dataset is based upon historical maintenance records. Finally, and after the training phase, newly arrived JSON messages are passed through each autoencoder that form a parallel connected complex in order to be classified into one out of the three supported labels. On the basis of the achieved classification accuracy, the remaining useful life is determined, as mentioned in the previous section, and exported to the user of the system. During the experimentation stage, the accuracy of the results was cross-validated with the actual maintenance records of the case owner, as discussed in the following section.

## 5. Case Study

**Figure 3:**
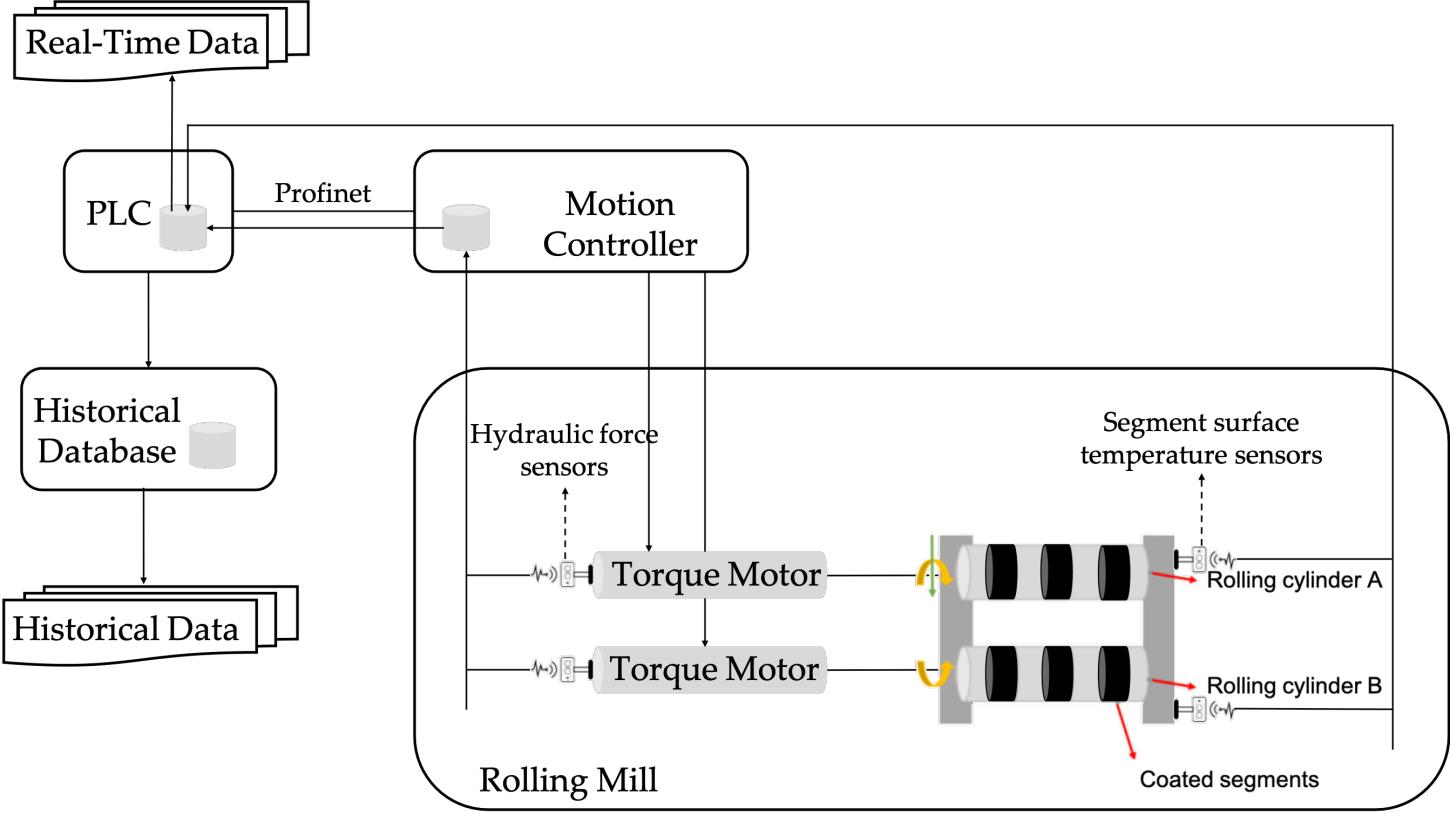
Rolling mill machine.

In order to evaluate the potential of the proposed concept, the implemented prototype was tested in a case study related to the steel production industry. In particular, the data were derived from a rolling mill machine used for metal bars production. A high-level diagram of the machine components and its connectivity is illustrated in Figure 3. Sensor values were stored to a local database in the motion controller and then transferred to a Programmable Logic Controller (PLC) database. Finally, data values were stored in the historical database. Real-time data were transmitted via communication channels from the PLC database to the PC for the RUL prediction.

Different geometrically coated segments were attached to the rolling cylinders. The segments were used in order to form the metal bars by applying force. The rolling mill had three top and three bottom segments with a wear-resistant coating. Until now, the coated segments of this rolling mill machine were scheduled to be replaced approximately every 10,000 products, or sooner in case of any unexpected damage. The time needed for the replacement of the coated segments was about two hours. Hence, the purpose, in this use case, was to turn preventive maintenance into predictive and anticipate the behavior of the segments.

Equipment conditions were monitored using multiple sensors on the machine that counted twenty-seven (27) factors for the equipment and its environment.Real-world data tend to be inconsistent, noisy, and incomplete, leading to a low quality of models built on them. In order to prepare these raw data to meet their requirements, pre-processing steps are of great importance [54]. The pre-processing of the data was accomplished via a separate software module. In particular, this system took as input JSON files with twenty-seven (27) features every forty-five seconds (45 s), and each sensor collected data every five milliseconds (5 ms). Afterwards, the zero and missing values were removed as fault values of the sensors. The subtraction of these values did not affect the results due to the big sample that was provided for each timestamp. Finally, as a result of the preprocessing phase a dataframe was exported.

However, the identification of the correct set of parameters that may be associated to potential failures of the production equipment is a challenging task. Hence, the values of the dataframe were plotted and visually examined against factory expert’s knowledge and maintenance records to identify patterns in order to make a first selection of the important features. Thus, according to data visualization, machine-related studies, and scientific dissertations, the critical values that determine the rolling mill’s degradation process were identified [12,55]. The parameters used for this analysis were surface temperature and hydraulic force for both cylinder A and B, Table 1.

These data values were processed as time-series data because tools like Discrete Wavelet Transform (DWT) and Fast Fourier Transform (FFT) have not been preferred for this case. From our analysis of the data, the oscillating components likely will not reveal any regular behavior in terms of frequency because the oscillations of the features used, the temperature, and the hydraulic force did not appear to present any periodicity [56].

Before setting up the three LSTM-autoencoder models, three datasets were created, that represent the three different situations of the machine, defined according to the previous segment’s exchanges records. Each dataset included about 200,000 values. Then, in order to define, train, and test data for each LSTM-autoencoder a simple split was performed; 90% of the first part of the dataset was the train data, thus approximately 180,000 values, and the remaining 10% consisted of the test data corresponding to approximately 20,000 values. Subsequently, train and test data were normalized in ranges from 0 to 1 enabling a faster training of the neural networks.

**Figure 4:**
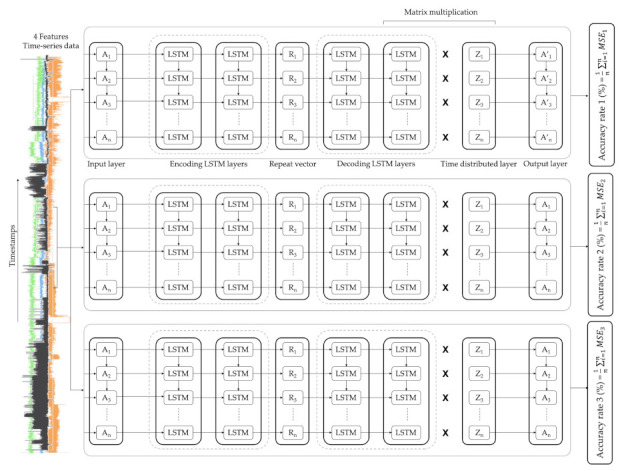
LSTM-autoencoder architecture set.

As illustrated in Figure 4, the architecture of each LSTM-autoencoder included, initially, an input layer where the size depends on the number of features selected, in this case four (4), as four (4) features were chosen for this model. Then, the first encoding LSTM layer read the input data and output sixteen (16) features with one timestep for each. The second encoding LSTM layer red the 1 × 16 input data from the first encoding LSTM layer and reduced the feature size to four (4). The output of this layer is a 1 × 4 feature vector. A repeat vector replicated the feature vector and prepared the 2D array input for the first LSTM layer in the decoder. Then, the first decoding LSTM layer read the 1 × 4 input data and output four (4) features with one (1) timestep for each. The second decoding LSTM layer read the 1 × 4 input data and output 16 features with one (1) timestep for each. Then, a time distributed layer took the output and created a 16 × 4 (number of features outputted from the previous layer x number of features) vector. Finally, a matrix multiplication between the second decoding LSTM layer and the time distributed layer outputted the 1 × 4 output.

## 6. Results

Performance evaluation followed, exploiting data from three (3) months of machine operation. Datasets were created, as mentioned previously, from historical maintenance records from the factory and the equipment state they represent.

The architecture of the implemented LSTM-autoencoder, as described in the previous section, including the layers of the network created, the number of parameters (weights and biases) of each layer, and also the total parameters of the model, are presented in Table 2.

Parameters affect the processing complexity of the network [57]. However, the total number of trainable parameters in each LSTM-autoencoder network was 3.236, presenting satisfying results for the specific dataset.

After the training, accuracy, recall, precision, specificity and F1 score metric values of the three LSTM-autoencoders were calculated after feeding them with test data [58,59]. However, the model’s performance was evaluated mostly based on accuracy because the other metrics presented small differences between the different test datasets, as presented in Table 3, Table 4, Table 5 and Table 6.

In order to identify the ideal number of epochs and batch sizes in this use case, several experiments were conducted, and the best accuracy rate results are presented in Table 7.

As a result, each LSTM-autoencoder was trained with the number of epochs and batch sizes presented by the accuracy rate. The initial-state LSTM-autoencoder presented better accuracy results after training with forty (40) epochs, while the intermediate-state LSTM-Autoencoder presented higher accuracy results after training within seventy (70) epochs. Finally, the good-state LSTM-autoencoder presented better accuracy results after training within a hundred (100) epochs.

Each dataset was the input to an LSTM-autoencoder. The reconstructed values presented a smaller reconstructed error for the LSTM-autoencoder that was trained with the data representing the same state to the input. Consequently, they represent a bigger accuracy rate(%), Table 8, where the first two columns refer to the actual states of the monitored equipment at the specific timestamps, while next to them is provided the accuracy rate generated by each one of the three LSTM-autoencoders for the corresponding timestamps.

The difference, though, between those three health statuses was not so big, but it was enough in order to characterize and provide a label for the type of the data and the status of the component’s health accordingly. During analysis, this observation was characterized as acceptable and logical, as the factory proceed to conduct maintenance activities on the component before it was completely damaged. Thus, the health status of the coating was not bad during the maintenance, and as a result the remaining useful life can be extended.

Assuming that the everyday production rate is constant, and that the fatigue of the machine is proportional to the number of produced trailing arms, the time interval between two maintenance events is determined by the working time of the machine and, consequently, by the number of created products. To this end, the fatigue rate was considered constant, and according to Table 8 the equipment could be functional approximately three (3) to five (5) days more. When the accuracy rate (%) between the bad state of the current condition and the condition where break down occurred was bigger than 10%, Table 8, then the RUL was three (3) days, and for each increase by 10% the RUL value increased by 2 days.

Considering the actual preventive maintenance plan for a period of approximately three (3) months, a comparison was made between the actual maintenance activity and the one suggested by the proposed approach, Table 9. In particular and regarding the aforementioned table, the first two columns indicate the day the new equipment was mounted/unmounted, while under the cause column the reason is stated. Next, the unmounting date as suggested by the proposed method is included in the next column, while in the last is provided an estimation on the additional days the equipment could remain mounted and operational, also based on the proposed approach.

According to the results, the LSTM-autoencoders predicted equipment break down one (1) day before the actual event. Additionally, the network results show that the equipment was still in a healthy state at the time of preventive maintenance activities.

Consequently, in a period of one (1) year, as preventive maintenance activities take place every sixteen (16) days, the equipment could gain (on average) approximately ninety-six (96) more days of life and 22.22% reduction in preventive stoppages.

## 7. Conclusions

In conclusion, this study discussed a deep learning approach based on LSTM-autoencoders for assessing the condition of a hot rolling milling machine and estimating its RUL value. Three separate autoencoders have been trained, one per label considered in the current study. Real-world data were used for training and testing a prototype implemented in Python. The prototype was tested in a real-world case study with the results presented in this study. However, additional experiments are required to further test its performance and accuracy with a larger dataset of proper data quality in a greater period.

From the results, redundant and preventive stoppages in the production line can be reduced, at the same time decreasing the cost of maintenance operations. The main limitation of the proposed approach is that the use of multiple neural networks to identify the status and the RUL at higher resolution can be very difficult, as the system may predict fault classifications and may not be able to recognize neighbor states. Another limitation of this approach emerges from the need of maintenance records for labeling the datasets and the need of large amounts of proper quality data with maintenance events such as component breakdowns. These kinds of data may not be easily available in industrial practice due to the significance of preventive maintenance in order to avoid any important failure of the equipment.

Apart from further experimentation, one of the authors’ next steps would be to evaluate the performance of the proposed approach against other machine learning methods. Future work will focus on evaluating the proposed concept in consideration to real-world production and maintenance plans. DWT and FFT will be examined for better data analysis, as additional parameters that may affect the segment wear, such as pressure and product position, should be tested. Additionally, the performance of the proposed algorithm could be improved by optimizing the hyperparameters of each network. A comparison with other ML methods will take place in terms of parametric complexity, training accuracy, prediction accuracy, and training speed. Furthermore, a time-series forecasting approach will be tested based on the transformer architecture or on GRU networks instead of LSTMs. Finally, a maintenance scheduling application will be developed including both the LSTM-autoencoder and transformer architectures.

## Figures and Tables

**Table 1:** Features.

Feature Name	Feature Value	Feature Description
Cylinder A segment surface temperature	Celsius (°C)	Surface temperature of cylinder A
Cylinder B segment surface temperature	Celsius (°C)	Surface temperature of cylinder A
Cylinder A hydraulic force	Kilonewton (kN)	Force of cylinder A on trailing arm
Cylinder B hydraulic force	Kilonewton (kN)	Force of cylinder B on trailing arm

**Table 2:** Number of trainable parameters.

Layer	Type	Output Shape (Timesteps × Features)	Parameters
input1	InputLayer	1 × 4	0
lstm1	LSTM	1 × 16	1344
lstm2	LSTM	1 × 4	336
repeatvector1	RepeatVector	1 × 4	0
lstm3	LSTM	1 × 4	144
lstm4	LSTM	1 × 16	1344
timedistributed1	TimeDistributed	1 × 4	68
Total parameters:			3.236
Trainable parameters:			3.236
Non-trainable parameters:			0

**Table 3:** LSTM-autoencoders recall metrics.

Historical Maintenance Records		Recall Metrics		
Equipment State	Dates	Initial	Intermediate	Bad
Initial	2019-12-24 T 01:01:49–2019-12-25 T 02:42:55	80.98%	81.23%	81.34%
Intermediate	2020-01-10 T 00:38:54–2020-01-10 T 23:01:24	81.97%	81.91%	81.91%
Bad	2020-01-13 T 00:05:47–2020-01-13 T 23:38:20	78.28%	78.31%	78.29%
Initial	2020-02-20 T 00:46:49–2020-02-21 T 22:38:45	87.82%	88.18%	87.82%
Intermediate	2020-02-25 T 01:20:54–2020-02-26 T 03:46:3	74.41%	74.44%	74.49%
Bad	2020-03-01 T 01:53:56–2020-03-01 T 21:37:48	65.62%	65.80%	65.60%
Initial	2020-03-02 T 01:00:14–2020-03-02 T 23:15:5	76.06%	76.23%	75.98%
Intermediate	2020-03-08 T 00:10:24–2020-03-08 T 22:46:33	73.48%	73.66%	73.61%
Bad	2020-03-16 T 09:13:47–2020-03-16 T 21:37:4	77.26%	77.20%	77.15%

**Table 4:** LSTM-autoencoders precision metrics.

Historical Maintenance Records		Precision Metrics		
Equipment State	Dates	Initial	Intermediate	Bad
Initial	2019-12-24 T 01:01:49–2019-12-25 T 02:42:55	79.86%	79.95%	79.90%
Intermediate	2020-01-10 T 00:38:54–2020-01-10 T 23:01:24	79.06%	80.12%	79.58%
Bad	2020-01-13 T 00:05:47–2020-01-13 T 23:38:20	78.10%	78.34%	78.43%
Initial	2020-02-20 T 00:46:49–2020-02-21 T 22:38:45	88.03%	88.07%	88.09%
Intermediate	2020-02-25 T 01:20:54–2020-02-26 T 03:46:30	71.47%	74.08%	72.07%
Bad	2020-03-01 T 01:53:56–2020-03-01 T 21:37:48	65.46%	65.78%	66.02%
Initial	2020-03-02 T 01:00:14–2020-03-02 T 23:15:53	74.35%	74.97%	75.29%
Intermediate	2020-03-08 T 00:10:24–2020-03-08 T 22:46:33	72.94%	73.49%	73.34%
Bad	2020-03-16 T 09:13:47–2020-03-16 T 21:37:48	72.21%	77.22%	77.26%

**Table 5:** LSTM-autoencoders specificity metrics.

Historical Maintenance Records		Specificity Metrics		
Equipment State	Dates	Initial	Intermediate	Bad
Initial	2019-12-24 T 01:01:49–2019-12-25 T 02:42:55	91.20%	90.98%	91.23%
Intermediate	2020-01-10 T 00:38:54–2020-01-10 T 23:01:24	90.16%	90.39%	90.41%
Bad	2020-01-13 T 00:05:47–2020-01-13 T 23:38:20	89.70%	89.92%	89.96%
Initial	2020-02-20 T 00:46:49–2020-02-21 T 22:38:45	85.74%	85.68%	85.64%
Intermediate	2020-02-25 T 01:20:54–2020-02-26 T 03:46:30	85.35%	85.84%	85.98%
Bad	2020-03-01 T 01:53:56–2020-03-01 T 21:37:48	85.55%	85.63%	86.45%
Initial	2020-03-02 T 01:00:14–2020-03-02 T 23:15:53	86.79%	86.82%	87.92%
Intermediate	2020-03-08 T 00:10:24–2020-03-08 T 22:46:33	74.99%	75.31%	75.71%
Bad	2020-03-16 T 09:13:47–2020-03-16 T 21:37:48	75.85%	75.98%	76.63%

**Table 6:** LSTM-autoencoders F1 score metrics.

Historical Maintenance Records		F1 Score Metrics		
Equipment State	Dates	Initial	Intermediate	Bad
Initial	2019-12-24 T 01:01:49–2019-12-25 T 02:42:55	80.41%	80.58%	80.61%
Intermediate	2020-01-10 T 00:38:54–2020-01-10 T 23:01:24	80.05%	80.83%	80.29%
Bad	2020-01-13 T 00:05:47–2020-01-13 T 23:38:20	78.19%	78.32%	78.36%
Initial	2020-02-20 T 00:46:49–2020-02-21 T 22:38:45	87.92%	88.12%	87.95%
Intermediate	2020-02-25 T 01:20:54–2020-02-26 T 03:46:30	72.54%	74.25%	73.00%
Bad	2020-03-01 T 01:53:56–2020-03-01 T 21:37:48	65.54%	65.79%	65.80%
Initial	2020-03-02 T 01:00:14–2020-03-02 T 23:15:53	74.91%	75.46%	75.50%
Intermediate	2020-03-08 T 00:10:24–2020-03-08 T 22:46:33	73.13%	73.56%	73.42%
Bad	2020-03-16 T 09:13:47–2020-03-16 T 21:37:48	77.23%	77.21%	77.20%

**Table 7:** Accuracy results from testing the implemented autoencoders.

Accuracy (%)	Epochs100-Batch10	Epochs70-Batch10	Epochs40-Batch10
Initial State	93.99%	93.45%	98.91%
Intermediate State	95.08%	99.74%	94.21%
Bad State	99.44%	93.20%	89.48%

**Table 8:** LSTM-autoencoders accuracy rates.

Historical Maintenance Records		Accuracy Rates (%)		
Equipment State	Dates	Initial	Intermediate	Bad
Initial	2019-12-24 T 01:01:49–2019-12-25 T 02:42:55	90.09%	83.71%	85.51%
Intermediate	2020-01-10 T 00:38:54–2020-01-10 T 23:01:24	73.42%	82.28%	73.42%
Bad	2020-01-13 T 00:05:47–2020-01-13 T 23:38:20	82.43%	86.74%	91.21%
Initial	2020-02-20 T 00:46:49–2020-02-21 T 22:38:45	96.09%	72.91%	63.44%
Intermediate	2020-02-25 T 01:20:54–2020-02-26 T 03:46:30	75.31%	94.80%	80.15%
Bad	2020-03-01 T 01:53:56–2020-03-01 T 21:37:48	92.54%	82.59%	81.55%
Initial	2020-03-02 T 01:00:14–2020-03-02 T 23:15:53	93.22%	89.10%	88.05%
Intermediate	2020-03-08 T 00:10:24–2020-03-08 T 22:46:33	70.31%	87.80%	80.15%
Bad	2020-03-16 T 09:13:47–2020-03-16 T 21:37:48	89.65%	71.23%	70.41%

**Table 9:** Experimental Results.

Mounted	Unmounted	Cause	Suggested Unmounted Date	Days Gained
2019-12-23	2020-01-14	Broken	2020-01-13	-
2020-02-19	2020-03-02	Preventive maintenance	2020-03-04	approx 3
2020-03-02	2020-03-16	Preventive maintenance	2020-03-18	approx 5

## Data Availability

Data available on request due to privacy restrictions.
